# Endocrine disrupters: the new players able to affect the epigenome

**DOI:** 10.3389/fcell.2015.00037

**Published:** 2015-06-18

**Authors:** Lavinia Casati, Ramon Sendra, Valeria Sibilia, Fabio Celotti

**Affiliations:** ^1^Department of Medical Biotechnology and Translational Medicine, University of MilanMilan, Italy; ^2^Departament de Bioquímica i Biologia Molecular, Universitat de ValènciaValencia, Spain; ^3^Department of Pharmacological and Biomolecular Sciences, University of MilanMilan, Italy

**Keywords:** epigenetics, endocrine disruptors, polychlorinated biphenyls, androgen receptor (AR), histone demethylases, Jarid1b

## Abstract

Epigenetics represents the way by which the environment is able to program the genome; there are three main levels of epigenetic control on genome: DNA methylation, post-translational histone modification and microRNA expression. The term Epigenetics has been widened by NIH to include “both heritable changes in gene activity and expression but also stable, long-term alterations in the transcriptional potential of a cell that are not necessarily heritable.” These changes might be produced mostly by the early life environment and might affect health influencing the susceptibility to develop diseases, from cancer to mental disorder, during the entire life span. The most studied environmental influences acting on epigenome are diet, infections, wasting, child care, smoking and environmental pollutants, in particular endocrine disrupters (EDs). These are environmental xenobiotics able to interfere with the normal development of the male and female reproductive systems of wildlife, of experimental animals and possibly of humans, disrupting the normal reproductive functions. Data from literature indicate that EDs can act at different levels of epigenetic control, in some cases transgenerationally, in particular when the exposure to these compounds occurs during the prenatal and earliest period of life. Some of the best characterized EDs will be considered in this review. Among the EDs, vinclozolin (VZ), and methoxychlor (MXC) promote epigenetic transgenerational effects. Polychlorinated biphenils (PCBs), the most widespread environmental EDs, affect histone post-translational modifications in a dimorphic way, possibly as the result of an alteration of gene expression of the enzymes involved in histone modification, as the demethylase Jarid1b, an enzyme also involved in regulating the interaction of androgens with their receptor.

## Epigenetics: what are the mechanisms?

Epigenetics represents the programming of the genome to express the appropriate set of genes in a space- time specific way during life (Zhang and Ho, 2011). Epigenetic patterns are created during cellular differentiation by a highly programmed and organized process (Casati, 2013). However, epigenetic mechanisms are dynamic and responsive to the environment, especially during the critical periods of embryonic development and early life (Casati, 2013). The epigenome is constituted by a set of chromatin players, such as DNA methylation, histone modifications and miRNA expression, that dynamically interact to define a correct transcriptomic profile (Fleisch et al., 2012). The effect of the epigenetic regulation can be an extensive change in cell gene expression, as occurs in several instances of DNA methylation, or the fine modulation of specific genes (Casati, 2013). In this review we have briefly considered the three main levels of the epigenome.

## DNA methylation

The genomic distribution of methylated DNA sequences is defined “methylome”; the “methylome” it is able to modify itself in function of the environment or the developmental stage. DNA methylation involves the covalent addition of a methyl group at position 5 of the pyrimidine ring of cytosine in CpGs dinucleotides, called CpG sites (Lister et al., 2009). DNA regions rich in CpG sites are known as CpG islands (Bird, 2002). In the human genome 60–80% of 28 million CpG dinucleotides are methylated (Lister et al., 2009). Unmethylated CpG islands are targets of transcription factors to start transcription. By contrast, the CpG sequences in inactive genes are usually methylated to suppress their expression (Belzil et al., 2013). For some transcription factors, for example, AP-2, c-myc, CREB/ATF, E2F, and NF-kB, DNA methylation abolishes access to promoter binding sites. However, this action mechanism seems to be true only for a subset of transcription factors (Kulis and Esteller, 2010). Current evidences support a second mechanism in which DNA methylation patterns correlate with chromatin structure and function. Active regions, characterized by an open chromatin structure, where genes are expressed, are associated with hypomethylated DNA sequences, whereas hypermethylated DNA is packaged in a more compact and inactive chromatin (Razin, 1998; Casati et al., 2010). A number of different proteins able to bind specifically to methyl-CG has been identified and shown to perform critical roles in the regulation of gene expression (Buck-Koehntop and Defossez, 2013). These proteins contains methyl-CpG binding domains (MBDs) which are stretches of about 75 amino acid residues long that are evolutionary conserved. Generally, DNA methylation seems to be a starting step for establishing the inactive chromatin state. It is followed by an MBD protein association that, in turn, recruits further repressive epigenetic modification enzymes, such as histone deacetylase (Kulis and Esteller, 2010) (see next section). The chromatin compacts and gene silencing is achieved. For example, a specific protein, MeCP2 (methylcytosine-binding protein 2) binds directly to methylated CG but not to unmethylated CG and its binding produces a tightly packed close chromatin structure and transcriptional repression. The importance of MeCP2 is shown by the finding that mutant MeCP2 forms, unable to recognize methyl-CG, produce the Rett syndrome, a severe developmental disorder leading to mental retardation (Adkins and Georgel, 2011).

DNA methyltransferases (DNMTs) are the enzymes involved in DNA methylation, of which at least three functional DNMTs have been identified in eukaryotic systems. DNMT1 is involved in maintenance of methylation status during replication (it can methylate only the CG sequence paired with methylated CG) (Reik et al., 2001); DNMT2 is related to embryonic stem cells and potential RNA methylation (Clouaire and Stancheva, 2008); and the DNMT3 family consisting of two members, DNMT3a and DNMT3b, which are involved in *de novo* DNA methylation at CpG sites occurring during early embryogenesis and are essential for the mammalian development (Singh and Li, 2012).

## Histone modification

The basic repeating unit of chromatin, the nucleosome, consists of 146 bp of DNA wrapped around an octameric histone core formed by two copies each of histones H2A, H2B, H3, and H4 (Felsenfeld and Groudine, 2003). Histones beside possessing a definite structural function have a specific role in modulating the physical access of nuclear factors to DNA (Luger et al., 1997). Histones regulate the chromatin compaction degree: in this way they are able to regulate the transcriptional activity as well as transcriptional silencing (Kanherkar et al., 2014). How is it possible? It is now clear that post-translational modifications of charged aminoacids of histone tails that protrude from the nucleosome can alter chromatin conformation and create binding sites for transcription factors; in this manner they can play a direct regulatory role in gene expression (Felsenfeld and Groudine, 2003). There are a lot of histones post-translational modifications that involve mostly lysine, arginine, threonine and serine residues (Cheung and Lau, 2005; Casati et al., 2010). Among them, the modifications more extended are acetylation, methylation, phosphorylation, ubiquitination, sumoylation, and ADP ribosylation (Cedar and Bergman, 2009). It is therefore apparent that a very strong modulating activity can be produced by the many possible combinations of modifications that can occur on a variety of sites on histones (Cheung and Lau, 2005). Among all the post-translational modifications of histones, lysine methylation and acetylation of histones H3 and H4 (Fischle et al., 2003) are the best studied. Histone methylation is catalyzed by histone lysine methyltransferases (HKMTase), whereas histone acetyltransferase (HAT) and histone deacetylases (HDACs) regulate, respectively, the acetylation, and deacetylation of lysine residues (Szyf, 2009).

It is recognized that histone post-translational modifications can regulate DNA accessibility by two different, but not mutually exclusive, ways (Suganuma and Workman, 2011). In one model, post-translational modifications of histones directly modulate chromatin compaction states across changes on the physico-chemical properties of the chromatin at the modification sites, thereby altering DNA–histone and histone–histone interactions within the nucleosomes or between nucleosomes. For example, acetylation of lysine residues neutralizes positive charges of histones and affects the electrostatic interactions between positively charged histones and negatively charged DNA. In the second way, histone post-translational modifications generate signaling platforms to recruit a variety of chromatin-binding proteins that recognize specific patterns of modifications on histones (“readers” or “effectors”), which subsequently lead to downstream cellular programs such as transcription modulation. Different protein domains have been identified that can recognize specific histone modifications, although they appear to be more flexible than the enzymes that create the modifications (Patel and Wang, 2013). For example, bromodomains recognize specifically acetyl-lysine residues on histones, whereas chromodomains bind methylated lysines, and tudor domains bind methylated arginines. Many evidences have revealed that histone post-translational modifications can act as a heritable “code” (so-called “histone code”) that can be passed during cell division to the progeny. Histone post-translational modifications could therefore permit the inheritance of phenotypic features independent of the DNA sequence. Given their involvement in fundamental cellular processes, dysfunction of histone post-translational modifications is found in diverse human diseases, particularly in cancer (Chi et al., 2010).

## RNA interfering

The third epigenetic mechanism is the post-trascriptional RNA induced silencing mediated by small, non-coding RNAs which down-regulate or suppress expression of specific genes. The silencing process is operated by microRNAs (miRNAs) and by short interfering RNAs (siRNAs); they are both 20–30 nucleotide-long double-stranded RNA molecules, encoded by their own set of cellular genes (miRNAs) or introduced into the cell from outside sources (siRNAs), e.g., virus (Carthew and Sontheimer, 2009). Although microRNAs only represent 1% of the genome, they have been estimated to mark 30% of genes (Lewis et al., 2005). These RNAs can act as switches and modulators, exerting extensive influence within the cell, fine-tuning the gene expression in specific cell-types during development as well as in pathological conditions (Baer et al., 2013). MicroRNAs have also been shown to play a role in cancer inhibition, apoptosis, cellular proliferation and cell movement suggesting that they can be used to supply an epigenetic cure to cancer (Kala et al., 2013).

## Epigenetics and environment: focus on the environmental factors able to shape the epigenome

The epigenetic changes might be produced by the environmental condition during the prenatal and early life and might influence the susceptibility to develop several diseases, from cancer to mental disorder during the entire life span (Foley et al., 2009). The epigenome integrates the informations present in the genome with the environmental cues, establishing the trascriptomic profile typical for each cell type to define its functional identity. The epigenome characterizes the capability of an organism to adapt and evolve regulating the characteristics or phenotypes developed in response to environmental cues (Rivera and Ren, 2013). The most studied environmental cues able to affect the epigenome are diet, child care, smoking, infections, wasting and environmental pollutants, especially endocrine disrupters (EDs) (Casati et al., 2010).

## Prenatal life

Gestation represents a very sensitive period in epigenetic remodeling and a lot of scientific evidences underlined the importance of parental influences on the offspring epigenome. Maternal health can determinate childhood development and adult health condition, defining the susceptibility to develop a disease during the adult life (Kanherkar et al., 2014). In particular, fetal programming expresses the way by which the uterine environment affects the fetal molecular development through epigenetic mechanism (Schulz, 2010).

One example is the influence of maternal diet and war stress on offspring epigenome exemplified by the famous “Dutch famine birth cohort.” It consists of more than 2000 singletons who were born between November 1943 and February 1947 in Amsterdam and systematically followed up since 1996 (El Hajj et al., 2014). Under the Nazi embargo of the Western Netherlands in 1944, pregnant women were under a severe nutritional restriction (El Hajj et al., 2014). Individuals who had been exposed to malnutrition and stress, during their early embryonic development, exhibited an increased risk for metabolic, cardiovascular and other complex diseases, schizophrenia, and accelerated cognitive aging (Schulz, 2010). More than 60 years after early gestational exposure, the Dutch famine individuals showed a subtle hypomethylation of the imprinted *IGF2*-*H19* locus, compared with their unexposed siblings (Heijmans et al., 2008). A follow-up study on the same cohort reported alterations dependent on sex and exposure length, in the DNA methylation status of several imprinted and non-imprinted genes in blood cells (Tobi et al., 2009). Likewise in experimental animal models as much diet as stress conditions of the mother affect the epigenetic signature during the fetus development (Kanherkar et al., 2014). In this sense, Barua and colleagues have shown that a maternal folic acid supplementation induces in the offspring a different global DNA methylation profile from that in the offspring of mice which received a low folic acid dosage (Barua et al., 2014). Specifically, a distinct DNA methylation status was observed on genes associated with autism spectrum disorder (ASD) pathogenesis (Barua et al., 2014). Furthermore, paternal influences can also affect the epigenome of the offspring. It has been shown, in animal models, that the as much the alcohol consumption as exposure to toxic chemicals, such as chromium chloride and vinclozolin, by the paternal progenitors affect DNA methylation in germinal cells (Stouder and Paoloni-Giacobino, 2010). Similarly, subjecting male mice to folate deficiency resulted in an alteration of sperm function related to the differential DNA methylation in comparison to control mice (Leonard et al., 2004; Lambrot et al., 2013). As well, the male offspring of such mice deficient in folate also showed an altered transcriptomic profile in comparison to the offspring from control mice with a normal folate supplement (Lambrot et al., 2013).

## Perinatal influences

A wide variety of environmental effects play an important role after birth. Particularly important appears to be the interplay between epigenetics and social influences. Environmental experiences, specially early life adversities, as low maternal care in rats, produce increased promoter DNA methylation of the glucocorticoid receptor (GR) in the hippocampus causing differences in its expression (Weaver et al., 2004). Such gene expression alteration results in blunted negative feedback inhibition by glucocorticoids and a heightened stress response that continues into adulthood (Weaver et al., 2004). Epidemiological data suggest that the effects of early life adversity involve many genes and are not limited to rodents (McGowan et al., 2008). In post-mortem hippocampal samples from humans who were abused in childhood, the promoters of ribosomal RNA (rRNA) and GR genes were found hypermethylated in correlation with their low expression (McGowan et al., 2008, 2009).

## Adult life

During the adult life, environment is able to shape the epigenome and there are many factors that can affect it such as diet, caloric restriction, alcohol consumption, and environmental pollutants. Several studies have shown how diet affects DNA methylation patterns (Jennings and Willis, 2015). Since foods are able to alter epigenetic expression, nutrients extracted from the diet could be utilized to influence disease susceptibility.

## Nutraceuticals

Micronutrients such as folate, retinoic acid, selenium compounds, polyphenols found in green tea, apples, coffee, black raspberries, and also other dietary sources containing genistein, soy isoflavones, curcumin, and resveratrol are able to influence epigenetic mechanisms and could be considered chemopreventive agents (Gerhauser, 2013).

One of the most studied epigenome modifiers is the phytoestrogen genistein, a bioactive isoflavone found in soy products, which is able to modulate the activity of DNA methyltransferases (Zhang and Chen, 2011). Genistein seems to affect tumorigenesis through epigenetic regulations, both histone methylation and DNA methylation (Zhang and Chen, 2011). Particularly, genistein appears to act, by modulating chromatin configuration and DNA methylation, activating tumor suppressor genes and affecting cancer cell survival (Zhang and Chen, 2011).

The polyphenol epigallocatechin-3-gallate (EGCG), present in green tea, is another example of micronutrient with antioxidant and chemopreventive properties (Fang et al., 2007), for which has been shown to slow down the carcinogenesis (Fang et al., 2007). The molecular mechanism of how EGCG works, inhibiting cancer cell growth, seems to be similar to that of other nutraceuticals such as soy genistein, and it involves DNA methylation regulation on key genes promoting positive epigenetic effects. Other bioactive compounds also considered as nutraceuticals are the sulfopropanes, present in and green tea and cruciferous vegetables, which have been associated with lower risk of cancer and other age-related diseases (Tollefsbol, 2014). It has been demonstrated that these compounds, in fact, have the capability to revert an aberrant epigenetic pattern (Tollefsbol, 2014).

## Environmental chemicals

Environmental pollutants, such as pesticides, are able to induce changes of DNA methylation in adults and also influence the susceptibility to different pathologies in offspring exposed during prenatal and early life (Kanherkar et al., 2014).

Widespread environmental contaminants belonging to heavy metal category, such as nickel, arsenic and cadmium disrupt normal histone acetylation and DNA methylation patterns, and have been related to different pathologies including tumorigenesis, neurological disorders, and autoimmune diseases (Leonard et al., 2004). A mode of action for these compounds has been hypothesized and may involve the fact that metals stimulate free radicals production inducing epigenetic alteration (Babar et al., 2008). For example, since S-adenosyl methionine (SAM), the universal methyl donor for methyltransferases (including DNMTs) is involved in arsenic detoxification (by methylation), arsenic exposure decreases the DNMTs activity but also, as has been shown, down-regulates DNMT gene expression (Reichard et al., 2007). Moreover, arsenic exposure induces hypermethylation of tumor suppressor genes (Jensen et al., 2008), disruption of histone acetylation (Hou et al., 2012), and up-regulation of miRNAs expression (Marsit et al., 2006). Likewise, nickel is able to stimulate DNA methylation of tumor suppressor genes (Lee et al., 1995), to condense chromatin and to affect histone acetylation, which is accompanied of gene silencing; these effects finally lead to cell transformation (Zhang and Zhu, 2012). It is worth noting, as a possible mechanism of action, that a study has shown that Ni binds to N-terminal tails of histone H4 and, promoting a secondary structure with organized side-chain orientation, decreases the ability of histone acetyltransferase to recognize and bind to the histone tail and thus affects the ability of the enzyme to further modify the lysine residues (Zoroddu et al., 2010).

## EDs: the new players able to affect the epigenome

There are environmental xenobiotics that can interfere with the normal development of male and female reproductive systems of wildlife and experimental animals, and very probably of humans, disrupting endocrine axis in adulthood. These compounds are defined as endocrine disrupters (EDs). The exposure to EDs plays a key role on the epigenome shaping of many aspects of the endocrine function (Casati, 2013). The evidences present in the literature indicate that EDs can affect the different levels of epigenetic control and in some cases can act transgenerationally, if the exposure to EDs occurs during the prenatal and early life. There are several EDs which can act on epigenome in multiple ways (Casati et al., 2012). Several enzymes involved in epigenetic key processes of regulation of the endocrine system, such as histone-modifying enzymes, are altered either directly in their catalytic power or in their expression levels by EDs (Casati, 2013). It is also known that nuclear steroid receptors interact with histone-modifying enzymes to regulate gene transcription and chromatin compaction (Leader et al., 2006). Interestingly, histone demethylases (the enzymes responsible of the removal of the methyl groups from histones) take part in protein complexes together with steroid receptors, in particular with the androgen receptor (AR), facilitating the transcription of their target genes (Gao and Alumkal, 2010). Among the known EDs vinclozolin (VZ), a fungicide with antiandrogenic activity, and methoxychlor (MXC), an organochlorine pesticide actively metabolized into a derivative with a potent estrogenic activity, promote epigenetic transgenerational effects. VZ administerd during gestation promotes a male germline epigenome reprogramming, which probably induces trans-generational adult-onset diseases, disrupting DNA methylation patterns in sperm of the F1 generation of animals lasting at least up to F3 generation. MXC exposure in female rats, is also able to produce differentially DNA methylated regions (DMR), termed epimutations, in sperm epigenome, however the increased disease incidence in F4 generation reverse (female) outcross offspring indicated that the transgenerational disease transmission is primarily through the maternal germline (Manikkam et al., 2014).

Therefore, environmentally induced epigenetic trans-generational transmission can involve either the male and/or female germ cells and in mammals occurs at the later stages of primordial germ cell migration and colonization of the fetal gonad and during the initial stages of gonadal sex determination (Skinner et al., 2013). It is possible that DMR occur after the erasure of DNA methylation prior to gonadal sex determination and then subsequent re-methylation in a sex-specific manner (Skinner et al., 2010). Interestingly, the transgenerational effects disappear gradually from F1 to F3. Transmission of the altered germline epigenome (DNA methylation) to subsequent generations in an imprinted-like manner produces an altered epigenome and transcriptome in all cell types and tissues that develop from the maternal or paternal germlines having an altered epigenome (Guerrero-Bosagna et al., 2012; Manikkam et al., 2014).

For example vinclozolin (VIN) exposure, tested at doses that are environmentally-relevant, produces testicular, ovarian diseases, reproductive anomalies but also affects sociosexual, anxiety, cognition, appetitive, and locomotor behaviors in several animal species including birds, rodents, fish, amphibians (Crews et al., 2012; Guerrero-Bosagna et al., 2013; Leon-Olea et al., 2014). The behavioral alterations may be partially attributable to VIN-induced alterations in gene expression (such as Gn-RH1, Esr1, Esr2, and Ar) in several hypothalamic nuclei, hippocampus, and striatum.

As mentioned above, one of the most studied EDs acting as an epigenome modifier is the phytoestrogen genistein, which is known to affect activity of DNA methyltransferases (Zhang and Chen, 2011). Moreover, modifications of the DNA methylation pattern in animals exposed to the synthetic estrogen diethylstilbestrol (DES) have been found (Casati et al., 2012). Likewise exposure to the pollutant bisphenol A (BPA), a plasticizer, also disrupt the DNA methylation pattern in agouti mice (Casati et al., 2012). Such BPA effect is reversible through food supplementation with methyl donor groups present in folate and genistein (Dolinoy et al., 2006).

Among the EDs there are the polychlorinated biphenyls compounds (PCBs) that are widely present in the environment (Casati et al., 2012). They were extensively used as dielectric and coolant fluids, i.e., in capacitors, transformers and electric motors (Colciago et al., 2009). Due to their toxicity and persistency in the environment, PCBs production was forbidden by USA in 1979 and by the Stockholm Convention on Persistent Organic Pollutants in 2001 (Colciago et al., 2006). PCBs are a group of 209 congeners with a broad spectrum of biological and toxic effects (Bonfanti et al., 2014). PCBs are classified as dioxin like (DL-PCBs), and non-dioxin compounds (NDL-PCBs) in function of their biochemical property (Casati et al., 2012). Many effects of DL-PCBs are mediated by the binding to the arylhydrocarbon receptor (AhR), a transcription factor present in many cell types of different animal species, including humans (Casati et al., 2012). The differential effects of DL- and NDL-PCBs present in the environment are indistinguishable *in vivo*, since animals and humans are exposed simultaneously to both classes and the final effect is cumulative (Casati et al., 2012). Although PCBs production was terminated in ‘70, they are still present in the environment and chronic low-level exposure to PCBs represents a significant public health issue (Casati et al., 2012). A lot of studies show that PCBs exposure causes endocrine, metabolic and behavioral effects in animals and humans (Colciago et al., 2009). Moreover, it has been shown that PCBs might alter directly the transcriptomic profile, particularly during development (Tabb and Blumberg, 2006). In addition, recent data show that PCBs are also able to disrupt epigenetic mechanisms (Casati, 2013). It appears that exposure of pregnant rats to a PCB mixture, throughout the period of gestation, reduces expression and activity of DNMTs in liver of the offspring (Desaulniers et al., 2009). Furthermore, our previously published results showed that the exposure to a reconstituted mixture of PCBs (PCB 126, 138, 153, and 180) during gestation induces the expression of Jarid1b (a histone H3K4me3 demethylase) and SIRT1 (a histone H4K16ac deacetylase), and consequently a reduction of H3K4me3 and H4K16ac levels, in the liver of the offspring (Casati et al., 2012). The same exposed animals were characterized by a decrease of AR gene and protein expression (Casati et al., 2012).

Since steroid receptors can act as cofactors of histone modifying enzymes, we analyzed in some details, the PCBs-AR-Jarid1b interaction (Casati et al., 2013). Therefore, we investigated: (1) how PCBs can affect the AR/Jarid1b interaction in the transcription of AR target genes; and (2) how PCBs affect AR/Jarid1b interaction in modulating the AR negative auto-feedback. Above all we considered: (a) the potentiating effect of Jarid1b on AR transactivation induced by PCBs (Casati et al., 2012); (b) the role of PCBs and Jarid1b in the transcriptional activity of different AR poly Q variants (AR isoforms with different transactivation capability); and (c) the molecular mechanism exerted by Jarid1b in the AR transactivation, and the interaction with the AR promoter (Casati et al., 2013). PCBs treatment, in a dose-dependent manner, promotes AR transcriptional activity although its effect is lower than that produced by the natural ligand dihydrotestosterone (DHT). DHT represents the active 5α-reduced testosterone metabolite, since possesses a higher affinity for AR than testosterone (Casati et al., 2013). Ligand binding studies have shown a specific and direct binding of several PCBs congeners to the ligand–binding domain of the AR protein (Portigal et al., 2002). Furthermore, Jarid1b is able to modulate the effects of AR-ligand interaction (Casati et al., 2013). The interplay between Jarid1b and AR on AR transactivation has been described in prostate cancer cells where Jarid1b is found up-regulated (Xiang et al., 2007). Likewise our previous *in vivo* studies have revealed that exposure to a mixture of PCBs stimulates the Jarid1b expression in the rat liver, and in its turn Jarid1b potentiates AR transcriptional activity (Casati et al., 2012). Moreover, in our more recent studies we have found that the overexpression of Jarid1b cotransfected with AR increases transcriptional activity induced by the treatment with DHT or PCBs in three different cell types: HEK293, and two neuronal cell lines, NSC34 and GN11 (Casati et al., 2013), indicating that the effect of the presence of Jarid1b on AR transactivation is not dependent on ligand or cell-phenotype. In spite of the described studies, the mode of action by which Jarid1b is able to modulate positively the AR transcriptional activity remains still uncertain. It is known that preservation of the enzymatic activity of Jarid1b is necessary for the increase of the AR transactivation, since the deletion of the JmjC domain, the demethylase catalytic center, eliminates the stimulation (Xiang et al., 2007).

PCBs-AR interaction is also modulated by differences in the structure of the AR gene found among individuals (Casati et al., 2013). It is known that AR transactivation is in part dependent on the length of a polyglutamine tract (polyQ, coded by a CAG repeat) located in the AR trans-activating region. The CAG repeat number in AR gene vary between 8 and 30 units in human populations, and thus the coded polyQ also is polymorphic in length (Ackerman et al., 2012). An inverse relationship has been found between the AR transcriptional activity and the length of poly Q repeat (Buchanan et al., 2004). Two recent reports by Bjork and coworkers show that PCBs have a CAG/PolyQ length dependent effect on AR *in vitro* (Bjork and Giwercman, 2013), and in some human prostatic cells (Bjork et al., 2011). In particular, PCB 153, present also in the reconstituted mixture used in our studies, has more pronounced effect on the *in vitro* AR transcriptional activity of short poly Q isoforms (Bjork et al., 2011). It is possible to hypothesize that Jarid1b-AR interaction affects the differential transcriptional activity of the AR isoforms, induced by PCBs, dependent on the interaction strength, which is lower for the long isoforms possessing a longer polyQ expansion (Casati, 2013). Moreover, Suzuki and coworkers have shown that the aberrant polyQ expansion potentiates the association between Rbp (Retinoblastoma Protein) and AR, and this association seems to attenuate the enrolment of HDAC1 (a histone deacetylase, class 1), which acts a potent transcription cofactor (Suzuki et al., 2009). It is possible that a similar mechanism could lead to the minor interaction shown in our studies for the longest isoform ARQ46, and consequently a low AR transactivation induced by PCB (Casati et al., 2013). On the contrary, the higher activation by the shorter AR isoforms seems to be mediated by a stronger interaction of this receptor with Jarid1b (Casati et al., 2013).

## A hypothesis: how the EDs could affect the epigenome? a link through steroid receptor and histone demethylases

**Figure 1:**
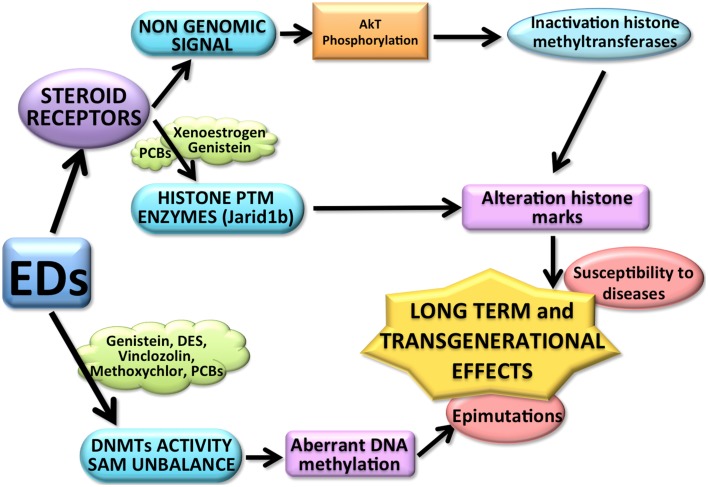
**The above diagram shows some of the possible ways by which EDs could affect epigenetic mechanisms**. EDs, for example PCBs, (through the interaction with the steroid receptors) might affect directly histone post-translational modification (PTM) enzymes but also inactivate the histone modification enzymes through a non-genomic signal pathways (for example Genistein). Both mechanisms can affect the histone modification balance, causing long term effects. EDs (such as DES, Vinclozolin, Methoxyclor, PCB, and Genistein) can also induce an aberrant DNA methylation pattern affecting not only the activity of DNMTs, but also changing the availability of S-adenosyl-methionine (SAM). The resulting aberrant methylome causes epimutations, possibly transmitted transgenerationally and might increase the susceptibility to develop diseases.

The intersection between nuclear receptor activity and the epigenetic apparatus has several implications in the mode of action of endocrine disrupters. Since there are many classes of ED, it is possible to hypothesize several ways by which ED could affect epigenetic mechanisms (see Figure 1).

Foremost it is possible that the alteration of DNA methylation activity induced by a single compound could be a common contributing factor to the dysregulation of several genes able to produce diverse phenotypic profiles (Anderson et al., 2012). The exposure to DES, an estrogenic ED, is a good example of this mechanism, since in animal studies the exposure to DES has been associated with a range of cancers, malformations of the genital track, and obesity (Newbold, 2011), probably related to alteration in DNA methylation pattern (Sato et al., 2009). Moreover, there are implications for direct or indirect long-term effects deriving from epimutations or aberrant epigenetic function (Anderson et al., 2012). For example an exposure *in utero* to some phthalates has been involved in the disruption of several steroidogenic pathway genes, contributing indirectly to malformations in offspring caused by the alteration of hormonal activity at critical developmental time points (Wilson et al., 2008). It is also possible that exposure to environmental pollutants affect histone methylation balance that indirectly produce long-term effects (Anderson et al., 2012). A permanent aberrant methylation might compromise the modulation of affected genes to future environmental cues, and thus increase susceptibility to develop disease during the entire life (Anderson et al., 2012). The rationale behind our hypothesis, linking epigenetic effects, EDs and steroid receptors, is that both short-term, indirect long-term and direct long-term effects might share a common etiology that involves, in part, nuclear-receptor mediated changes in histone methylation status (Anderson et al., 2012).

A paper from Wong and colleagues indicate that the perinatal exposure to EDs, and in particular xenoestrogens, increases cancer affecting the levels of DNA and histone methylation (Wong and Walker, 2013). Zhang and colleagues have analyzed the way by which xenoestrogens affect the epigenetic mechanism to reprogram the epigenome during the development (Zhang and Chen, 2011). These studies indicated that xenoestrogens induce non-genomic ER signaling activating PI3K/AKT pathways, resulting in AKT phosphorylation and inactivation of the histone methyltransferase EZH2, thus providing a direct relation to epigenome disruption (Zhang and Chen, 2011). Finally, it cannot be ruled out that non-genomic signaling could target also other epigenetic machinery components, suggesting this is a possible mechanism by which EDs could disrupt the epigenome (Wong and Walker, 2013).

On the other hand, according to our results, PCBs mediate a direct interaction between the histone demethylase Jarid1b and AR (Casati et al., 2013). The AR/Jarid1b binding on DNA of target gene seems to be allowed by the presence of Androgen Responsive Element (ARE) and by the presence of binding sites for the histone demethylases enzyme, Jarid1b (PLU1) and AhR (XRE), (Casati et al., 2013). As a matter of fact, in our previous studies, where the AR promoter DNA sequence was analyzed, we observed the presence of binding sites for Jarid1b (PLU1), some AREs, and XREs (Casati et al., 2013) [which at the same time suggests for a potential direct effect of the Jarid1b in modulating also the AR negative auto feedback (Vismara et al., 2009)]. In order to study the AR/Jarid1b interaction, we performed a series of gene reporter assays, where HEK293 cells were cotransfected with plasmids encoding for the luciferase gene, under the control of AR promoters with different lengths (long, intermediate and short), and the Jarid1b gene (Casati et al., 2013). Results showed that the presence of Jarid1b and, at least, two PLU1 binding sites are necessary for PCB-induced transactivation (Casati et al., 2013). We have hypothesized the involvement of Jarid1b as essential component for the interactions in the AhR-AR complex, occurring after exposure to PCBs, in particular in presence of DL congeners, since the responsive element XRE, ARE and PLU1 are concomitantly present on promoters of AR target genes.

Nevertheless, the relation between AR and AhR is complex and not fully understood (Kollara and Brown, 2010) and, to our knowledge, there is no data about a direct interaction between AhR and Jarid1b (Casati, 2013). It is possible to conclude that the AR modulation induced by PCBs involves AR-Jarid1b interactions. Further studies are needed to corroborate this hypothesis involving a delicate interplay between environment, epigenome and endocrine system (Casati, 2013).

## Conclusions

Perturbation of nuclear receptors and epigenetic players may be a common mechanism in the epigenome modification caused by EDs. As matter of fact, even if the target genes affected by the endocrine disrupters may differ, the underlying mechanism, such as the perturbation of the delicate interplay between the actions of different epigenetic participants seems to be a common action mode (Anderson et al., 2012). Further studies will be necessary to delineate in a more precisely way the mechanism by which EDs are able to modify the epigenome.

### Conflict of interest statement

The Reviewer Dr. Adriana Maggi declares that, despite sharing an affiliation with the authors, Dr. Lavinia Casati and Dr. Fabio Celotti, the review process was handled objectively and no conflict of interest exists. The authors declare that the research was conducted in the absence of any commercial or financial relationships that could be construed as a potential conflict of interest.
